# Salvianolic acid B from *Salvia miltiorrhiza* bunge: A potential antitumor agent

**DOI:** 10.3389/fphar.2022.1042745

**Published:** 2022-10-25

**Authors:** Sha-Sha Guo, Zhen-Guo Wang

**Affiliations:** ^1^ Key Laboratory of Theory of TCM, Ministry of Education of China, Shandong University of Traditional Chinese Medicine, Jinan, China; ^2^ Institute of Traditional Chinese Medicine Literature and Culture, Shandong University of Traditional Chinese Medicine, Jinan, China

**Keywords:** TCM, traditional Chinese medicine, salvianolic acid B, anticancer activity, apoptosis, mechanism

## Abstract

*Salvia miltiorrhiza* Bunge (Lamiaceae) is a perennial herb widely found in China since ancient times with a high economic and medicinal value. Salvianolic acid B (Sal-B) is an important natural product derived from *Salvia miltiorrhiza* and this review summarizes the anticancer activity of Sal-B. Sal-B inhibits tumor growth and metastasis by targeting multiple cell signaling pathways. This review aims to review experimental studies to describe the possible anticancer mechanisms of Sal-B and confirm its potential as a therapeutic drug.

## Introduction

*Salvia miltiorrhiza* Bunge (Lamiaceae) is a traditional Chinese herb that has long been used to effectively promote blood circulation and remove blood stasis (Bachheti et al., 2022; Huang et al., 2022). It has been widely used in China for thousands of years to treat various diseases, including coronary heart disease, myocardial infarction, angina pectoris, and atherosclerosis (Wang et al., 2017; Jia et al., 2019; Jin et al., 2022a). Scientists have researched and developed the Fufang Danshen Dripping Pill, a major innovation in TCM, to facilitate administration and absorption by humans (Hu and Wang, 2019). It is also the first compound TCM drug to pass second-phase United States Food and Drug Administration human clinical trials (Xu et al., 2014).

To date, dozens of lipophilic compounds of *Salvia miltiorrhiza* Bunge (Lamiaceae) have been identified, such as tanshinones IVI, and salvianphenol A (Jin et al., 2022b), as well as hydrophilic compounds including salvianolic acid A (Sal-A), salvianolic acid B (Sal-B), and protocatechuic aldehyde (Chang et al., 2022; Zhou et al., 2022). The most abundant hydrophilic compounds in Salvia are the phenolic acids (Hong et al., 2022; Jayusman et al., 2022), which are important for human health and are attracting increasing attention (Li et al., 2022). Sal-B exhibits anticancer activity in a variety of cell lines, including prostate, breast, liver, and head and neck squamous cell cancers.

This review summarizes advances in the extensive literature elucidating the antitumor effects and mechanisms of Sal-B compounds in various cancers, providing a reference for research and clinical application. Although several researchers have analyzed the important role of salvianolic acid B in the treatment and prevention of cardiovascular diseases, the anticancer properties of salvianolic acid have not been summarized. Therefore, this review aims to present the anticancer potential of salvianolic acid B and its mechanism of action to provide more information regarding this natural component of herbal medicine.

## Cancer and chemoprevention

The main cause of cancer development is the dysfunction of autophagy encoded by various genes, such as tumor suppressors, anti-apoptotic proteins, and growth factors, which allow unrestricted cell proliferation (Chen et al., 2022a; Shen et al., 2022). Patients with early-stage cancer are at risk of distant metastasis. Furthermore, multiple cellular and genetic alterations in the normal epithelium take many years to occur, leading to malignant changes. Therefore, the development of effective, less toxic, and affordable novel pharmacological agents to prevent cancer development is important.

Chemoprevention is a powerful method to prevent or slow down cancer progression (Acquaviva et al., 2022). In addition, many herbal medicines and related active compounds with potent anticancer activity, such as matrine and honokiol have been used as prophylactic agents. Current studies have shown that the anticancer properties of matrine are closely related to inhibition of proliferation and induction of apoptosis. Matrine induces apoptosis in U937 cells and K562 cells through a cytochrome c-triggered caspase-activated mitochondrial pathway (Zhou et al., 2014) but induces toxicity in mouse hepatocytes and its mechanism of action is dependent on reactive oxygen species (ROS) (Liu et al., 2020). Honokiol inhibits the growth and induced apoptosis in HNSCC cell lines and enhanced the growth inhibition and anti-invasive activity of erlotinib, a tyrosine kinase inhibitor (TKI) targeting EGFR (Leeman-Neill et al., 2010). However, these promising activities did not translate into clinical success despite these herbal active ingredients having tremendous potential medicinal properties.

## Salvianolic acid B

**FIGURE 1:**
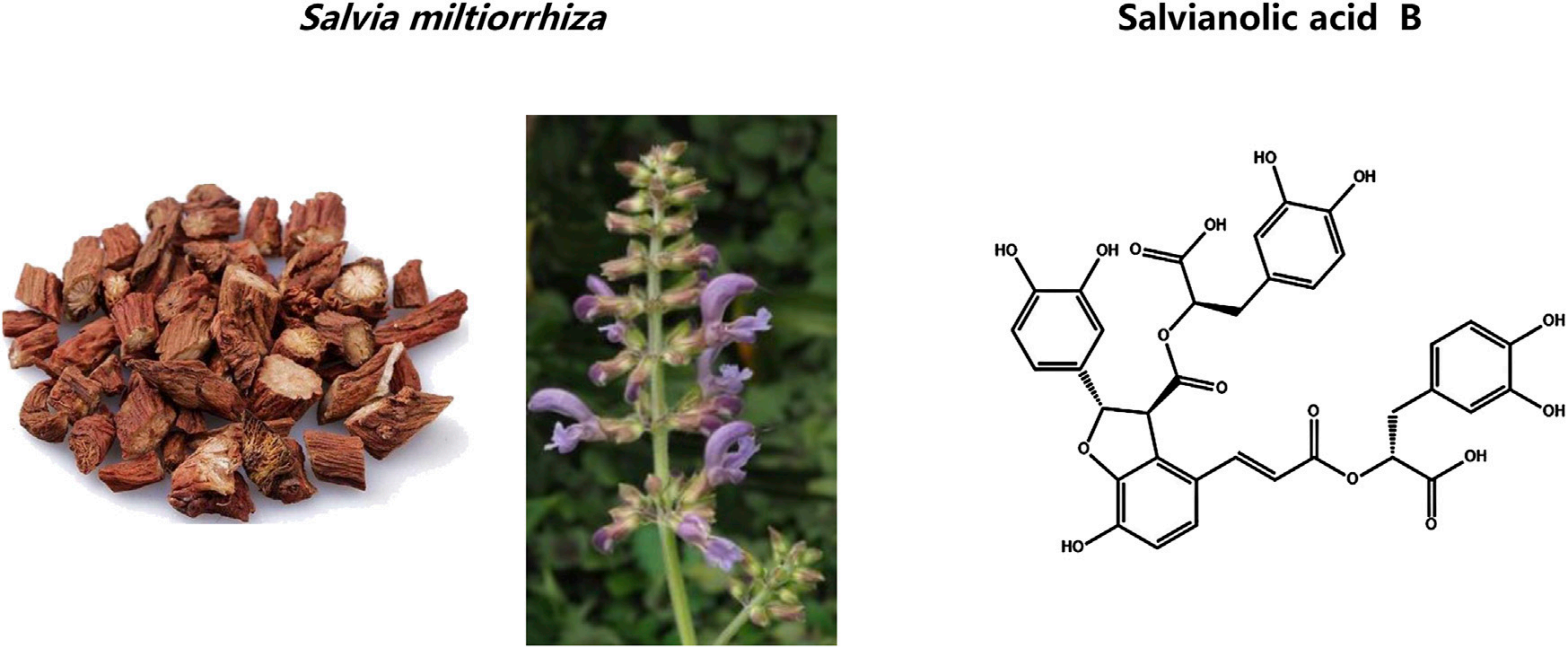
Chemical structures and natural sources of salvianolic acid B.

Sal-B is the most abundant and biologically active hydrophilic component of *Salvia miltiorrhiza*. According to the Chinese Pharmacopoeia (National Pharmacopoeia Committee, 2020), Sal-B is one of the important reference components for the quality standard of the traditional Chinese medicine *Salvia miltiorrhiza*. Sal-B contains seven phenolic hydroxyl radicals which have antioxidant activity (Wang et al., 2007) and its structure is shown in Figure 1. Sal-B is of increasing interest to researchers due to its preventive and therapeutic value for cancer as well as cardiovascular and neurodegenerative diseases (Liu et al., 2000). The mechanism is mainly due to its anti-inflammatory and antioxidant properties, regulation of apoptosis, and inhibition of platelet aggregation (Zhao et al., 2011). Sal-B also has therapeutic effects on a variety of cancers, such as lung carcinoma, breast cancer, oral squamous cell carcinoma, head and neck carcinoma, hepatocellular cancer, and glioma cancer cell lines (Kiemlian Kwee, 2016; Khan et al., 2020; Guan et al., 2022).

### Sal-B inhibits human hepatocellular carcinoma cell viability

Hepatocellular carcinoma is a major cause of mortality (Riaz et al., 2022; Xia et al., 2022), and hepatitis B and C viruses are major contributors to hepatocellular carcinoma. Unfortunately, most HCC patients are diagnosed at a late stage, thus surgery is not a treatment option (Khafaga et al., 2022; Wei et al., 2022). Traditional chemotherapy is important for cancer patients who are unable to undergo surgery but some current chemotherapy drugs have low response rates and side effects in hepatocellular carcinoma, so there is an urgent need to develop new drugs (Chun, 2022; Udoh et al., 2022). Recently, the anticancer effects of Sal-B have been demonstrated in human cancer cell lines and *in vitro* studies have shown that Sal-B induces cell death and promotes apoptosis.

**FIGURE 2:**
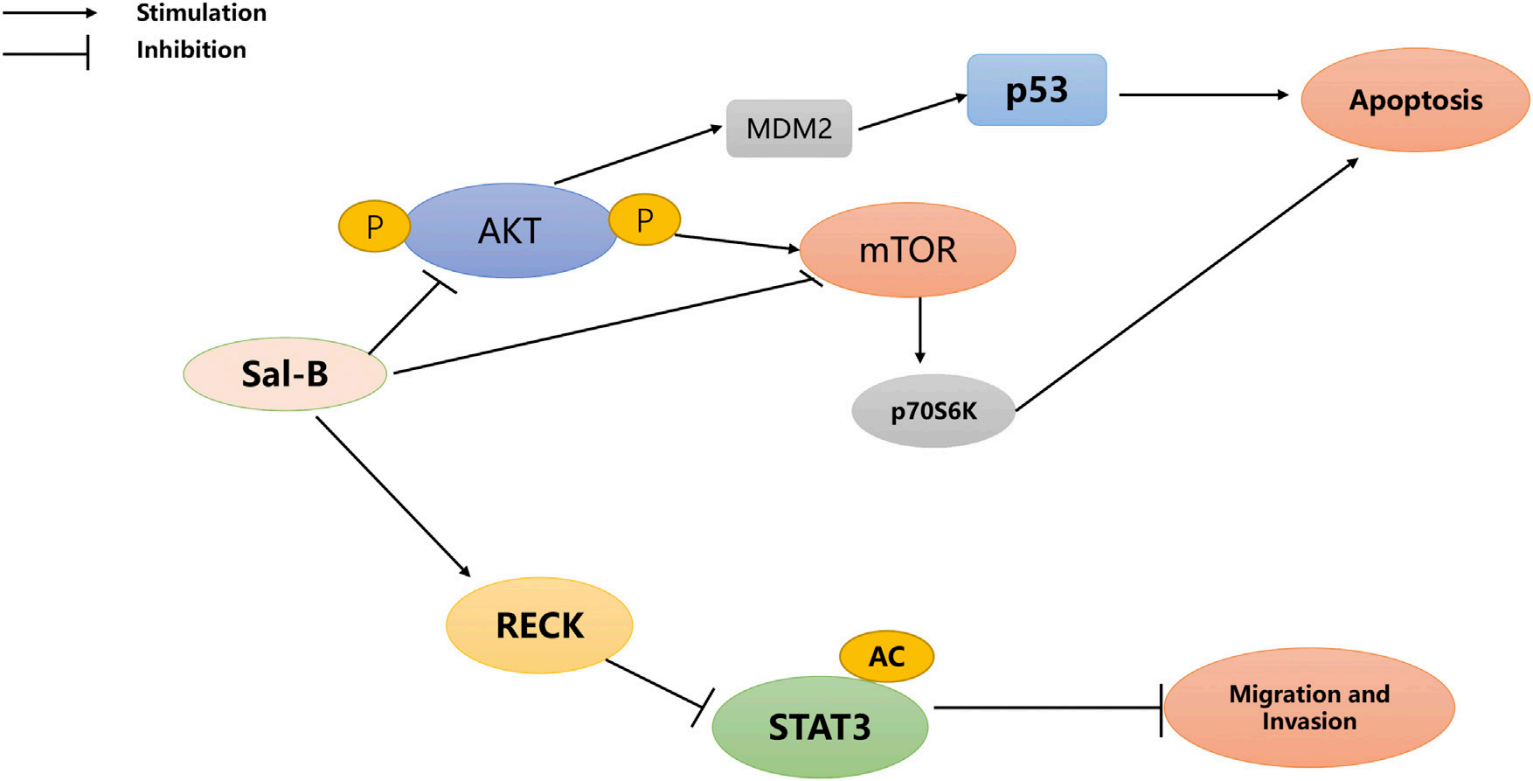
The anti**-**hepatocellular carcinoma pathway of Sal-B.

Fu et al. (2021) found a key factor in the ability of Sal-B to induce cell death is the promotion of autophagy and apoptosis in tumor cells. Moreover, Gong et al. showed that Sal-B-induced cell death was associated with AKT/mTOR signaling inhibition (Gong et al., 2016). Teng et al. suggested that Sal-B could be a potential anticancer agent for the treatment of HCC (Teng et al., 2021) (Figure 2). Furthermore, Hillmer et al. reported that Sal-B specifically bound to mortalin and increased the degradation of mortalin proteasomes through ubiquitination, thereby upregulating RECK, inhibiting STAT3, and finally inhibiting the migration and invasion of HCC cells (Hillmer et al., 2016).

(Reversion-inducing cysteine-rich protein with Kazal motifs, RECK; Signal transducer and activator of transcription 3, STAT3; protein kinase B, AKT; mammalian target of rapamycin, mTOR; murine double minute2, MDM2).

### A high potency against breast cancer

Breast cancer is the most common female cancer (Di Modica et al., 2022; Zhang H. et al., 2022), with a mortality rate second only to that of lung cancer, especially since the International Agency for Research on Cancer recently found that the mortality rate for breast cancer has now gradually surpassed that of lung cancer (Pearanpan et al., 2022; Yamashita and Kufe, 2022). Triple-negative breast cancer is highly malignant and difficult to treat (Hacking et al., 2022; Montalvo-Castro and Salinas-Jazmín, 2022). Although chemotherapy, radiation therapy, and systemic immunotherapy have led to longer survival, some patients with advanced breast cancer develop metastatic cancer (Gautam et al., 2022; Karn et al., 2022). Furthermore, the development and research of novel drugs to control metastasis remain a great challenge.

**FIGURE 3:**
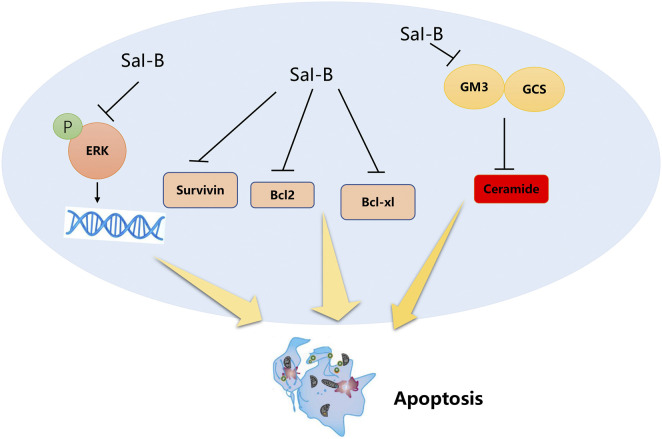
Sal-B inhibits Bcl-2, Bcl-xl, Survivin, glucosylceramide, and GM3 synthase expression, inducing ceramide-mediated apoptosis in breast cancer cells (Extracellular regulated protein kinases, ERK; B-cell lymphoma-2, Bcl-2; B-cell lymphoma–extra-large, Bcl-xl; ganglioside M3, GM3; glucosylceramide, GCS).

Sha et al. revealed that Sal-B can potently inhibit the growth of cultured triple-negative breast cancer cells *via* a ceramide-mediated pathway (Sha et al., 2018). Sal-B enhances apoptosis and reduces cell proliferation in TNBC by regulating ceramide glycosylase. Moreover, Sal-B has certain therapeutic advantages over current chemotherapeutic drugs (Murugan et al., 2016) as it is less toxic and dose-dependently induced apoptosis of MCF-7 in breast cancer cells (Katary et al., 2019). Furthermore, Qian et al. demonstrated that Sal-B reduced the level of the oxidative stress marker malondialdehyde and increased the plasma level of the antioxidant marker glutathione (GSH), thereby significantly reducing the tumor volume and increasing the median overall survival of solid cancer cells in mice (Qian et al., 2022). Ding et al. synthesized an FA-PEG-TiO2 nanocarrier to load Cur and Sal-B, as it acts synergistically with curcumin for an antitumor effect (Ding et al., 2016). The anticancer mechanism of Sal-B is summarized in Figure 3.

### Head and neck squamous cell carcinoma

Head and neck squamous cell carcinoma (HNSCC) are a scourge on human health (Johnson et al., 2020), and the main strategy to reduce its morbidity and mortality is the early diagnosis (Solomon et al., 2018). The chronic inflammatory microenvironment induces the transformation of normal cells into cancer cells (Yokota et al., 2020; Aulakh et al., 2022), the proliferation of tumor cells, and gene mutations (Park et al., 2022). Current treatments for HNSCC have limited therapeutic outcomes, thus it is important to develop novel pharmacological agents.

**FIGURE 4:**
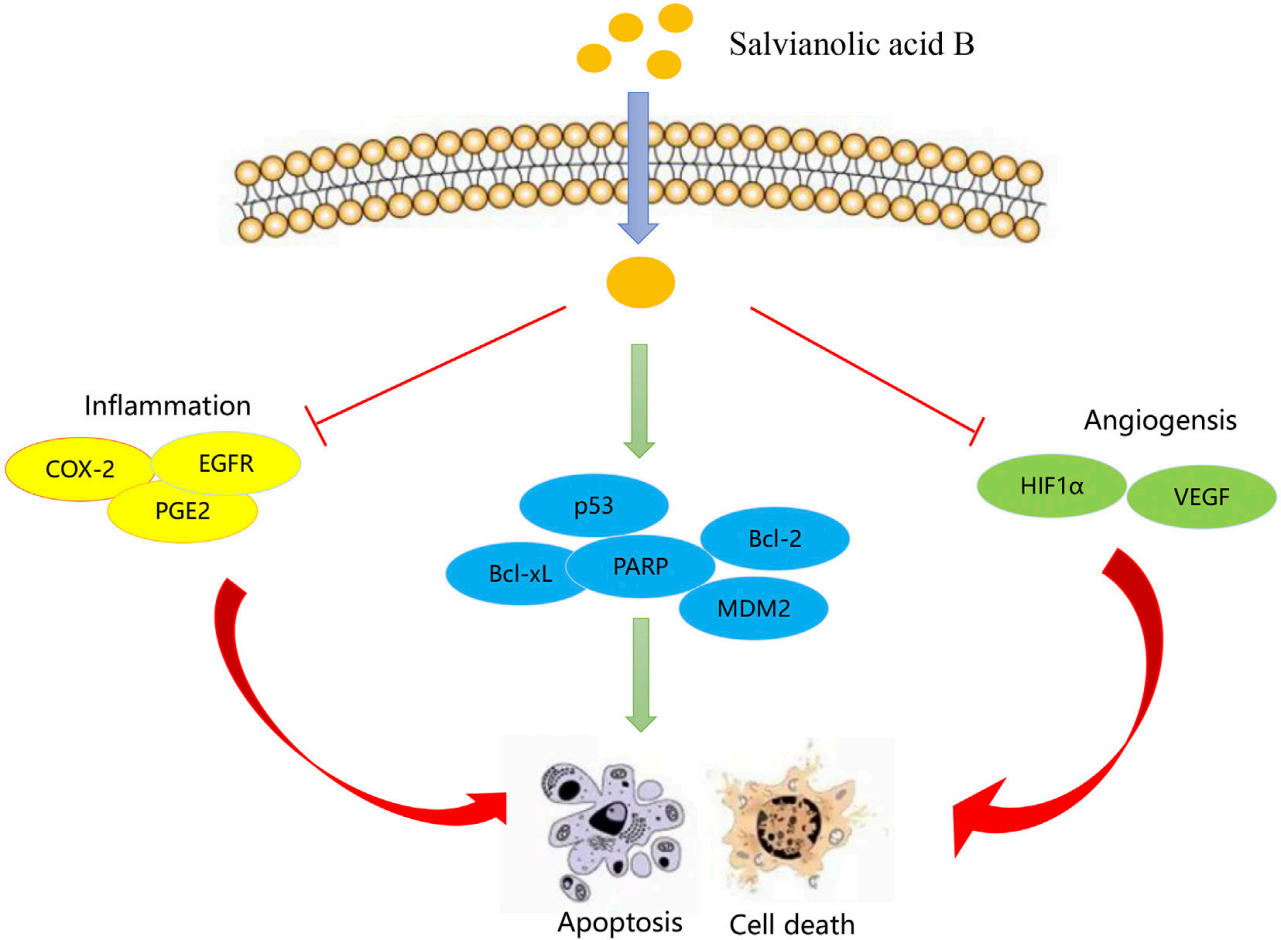
Molecular mechanism of Salvianolic acid B inhibiting HNSCC.

There is growing evidence that Sal-B is a promising chemotherapeutic agent for HNSCC (Kan et al., 2014). Cao et al. (2012) showed that Sal-B can block angiogenesis, thus preventing the transformation of normal epithelial cells into cancer cells and Zhao et al. (2010) found that Sal-B inhibited COX-2 expression in HNSCC cells of different origins. In addition, Sal-B can sequentially inhibit the COX-2/PGE2/EGFR pathway to induce apoptosis (Figure 4) (Hao et al., 2009). Phospholipid complex-loaded nanoparticles (PLC-NPs) encapsulating Sal-B serve as potential carriers in HNSCC (HN13, HN30) cells and Leuk1 cells, inducing apoptosis and cell cycle arrest, increasing the biological activity of Sal-B *in vivo* (Liu et al., 2007). Nano formulations encapsulate Sal-B within the backbone structure to enhance targeting and increase the drug bioavailability to improve the anticancer potential (Chen et al., 2022b; Liu et al., 2022). However, there are still few studies on the anti-head neck squamous cell carcinoma activity of Sal-B.

(Cyclooxygenase-2, COX-2; epidermal growth factor receptor, EGFR; Prostaglandin (PG) E2, PGE2; poly ADP-ribose polymerase, PARP; Hypoxia-Inducible Factor 1-Alpha, HIF-1α; vascular endothelial growth factor, VEGF).

### Inhibitory effect on oral squamous cell carcinoma

Oral squamous cell carcinoma (OSCC) has become a pressing medical problem worldwide and is the sixth most common malignancy (Panarese et al., 2019; Togni et al., 2022). Surgery and chemotherapy are the conventional treatments for OSCC, with chemotherapy being the most effective (Han et al., 2021; Siquara da Rocha et al., 2022). However, the severe side effects and resistance to chemotherapy are the biggest obstacles to treatment (Abdelmeguid et al., 2021). Therefore, there is an urgent need to find novel strategies and drugs.

A recent study reported the cytotoxic effect of Sal-B on OSCC by inhibiting tumor angiogenesis. Sal-B induced growth inhibition in OSCC cell lines but had limited effects on premalignant cells. Zhou et al. (2006) showed changes in the expression of 17 genes in Sal-B-treated OSCC cells, among which, HIF-1a, TNFα, and MMP9 were inhibited, whereas THBS2 was upregulated (Yang et al., 2011). Sal-B also inhibited the PI3K/AKT/HIF-1α signaling pathway and regulated abnormal glucose metabolism to prevent normal cell carcinogenesis (Wei et al., 2018).

### A therapeutic effect in non-small cell lung cancer

Lung cancer is one of the most prevalent and deadly malignancies (Herbst et al., 2018), with annually increasing morbidity and mortality rates (Pennell et al., 2019). Non-small cell lung cancer (NSCLC) is the most prevalent and aggressive type of lung cancer (Broderick, 2020) and comprises three main subtypes, large cell lung carcinoma, squamous cell lung carcinoma, and adenocarcinoma (Babuta et al., 2022; Oronsky et al., 2022). Currently, small molecule TKIs and immunotherapy are among the first-line treatments for NSCLC that have improved survival rates in some patients (Koulouris et al., 2022; Sinjab et al., 2022). However, the overall mortality and morbidity of NSCLC patients remain high, especially in advanced stages. Traditional Chinese medicine has significant efficacy in adjuvant therapy and improving the prognosis of NSCLC patients (Zhang R. et al., 2022).

Zhang S. X. et al. (2022) showed that Sal-B regulates β-catenin and E-cadherin, thereby inhibiting the migration and invasion of cancer cells and inactivating EMT. Sal-B down-regulated the expression of PKM2, LDHA, and GLUT1, affecting glucose uptake, lactate production, enolase activity, cellular ATP levels, and regulating cellular metabolic reprogramming in NSCLC (Chen B. et al., 2022). Yang et al. (2021) showed that Sal-B attenuates NSCLC metastasis through metabolic reprogramming independent of PKM2, revealing its therapeutic promise in the treatment of NSCLC. Han et al. concluded that Sal-B inhibited TGF-β1 and thus induced EMT and migration in A549 cells, hindered cell cycle progression, and promoted cell autophagy and apoptosis. In addition, Sal-B altered the phosphorylation of the MAPK signaling pathway and Smad2/3, especially Smad3 in the junctional region, leading to a decrease in the protein expression of PAI-1 in TGF-β1-stimulated A549 cells (Han et al., 2022). In conclusion, these results suggest that Sal-B has an inhibitory effect on NSCLC by blocking the activation of the MAPK and Smad2/3 signaling pathways, therefore, Sal-B may be a potential therapeutic candidate for NSCLC.

### Induces apoptosis in human glioma

Human malignant gliomas are aggressive and infiltrate the limited space of the intracranial cavity and are also common in the central nervous system (Chen et al., 2017; El Khayari et al., 2022). Glioblastomas are highly destructive malignant brain tumors (GBM; World Health Organization grade IV glioma) (Ludwig and Kornblum, 2017; Tsitlakidis et al., 2020), with a high proliferation rate and are highly aggressive. Current treatment is surgical resection, local irradiation, and conventional chemotherapy with temozolomide (TMZ) (Chandrasekar et al., 2022). According to recent studies, the overall median survival of GBM patients is short despite the use of multimodal therapy (Thakur et al., 2022).

Wang et al. (2013) found that Sal-B significantly reduces the viability of U87 cells in a dose- and time-dependent manner. Sal-B also enhances the production of ROS in U87 cells to induce apoptosis (Feng et al., 2022) and dose-dependently increases the phosphorylation of p38 MAPK and p53 (Byun et al., 2018; Chen et al., 2018). In conclusion, Sal-B could be a promising natural component in the treatment of hemangioma cells.

### Reduces drug resistance in gastric cancer cells

Gastric cancer also poses a great threat to human health (Correa, 2013; Guggenheim and Shah, 2013. The conventional clinical treatment remains surgery and chemotherapy (Cosma et al., 2022) but the development of resistance to chemotherapeutic drugs leads to failed recovery in most patients (Jelski and Mroczko, 2022; Sobczak and Kędra, 2022). Therefore, research and development of new drugs are key to improving drug efficacy and prolonging patient survival.

Chen et al. (2020) reported that Sal-B decreases tumor cell viability, promotes ROS production, induces apoptosis, as well as reduces migration, invasion, and EMT of AGS and AGS/DDP cells. Sal-B also regulates proliferation, EMT, and apoptosis to reduce the resistance to DDP *via* the AKT/mTOR pathway in DDP-resistant gastric cancer cells (Wang et al., 2021) (Figure 1). Therefore, Sal-B could be a potential antidrug-resistant agent to chemotherapy in gastric cancer. Tao et al. found that Sal-B exhibited superior inhibitory activities on neutrophil extracellular traps formation and significantly attenuated the levels of citrullinated histone H3 (citH3), a biomarker for neutrophil extracellular traps formation (Tao et al., 2018). Violi and Pignatelli (2015) demonstrated that Sal-B modulated the enzymatic cascade involved in NET formation and could disrupt NET formation at the earlier stage by blocking the activities of myeloperoxidase (MPO) and NADPH oxidase (NOX), respectively.

### Inhibition of cancer metastasis

Metastasis remains the greatest difficulty in cancer treatment and is associated with more than half of cancer-related deaths (Seyfried and Huysentruyt, 2013). EMT is the main factor involved in cancer cell metastasis, in which the most prominent role is played by the signals released by the mesenchymal cells that make up normal tissue or neoplastic tissue (tumor neointima) (Xu et al., 2005). In addition, matrix metalloproteinases, such as MMP-2/-9, induce metastasis in cancer cells (Mehner et al., 2014). Therefore, mediators targeting these essential metastases have the potential to prevent metastasis and overcome the invasiveness of cancer cells. Sal-B can block metastasis by inducing EMT markers such as E-cadherin, but additional studies should explore the effects of Sal-B on other EMT markers such as ZEB-1, ZEB-2, and TCF3 (Seyfried and Huysentruyt, 2013; Mehner et al., 2014).

### The comparison of different pathway in different cancers

Salvianolic acid B has been shown to inhibit a number of cancers. Although different cancers occur by different mechanisms, the treatment of some cancers has similar pathways. In the treatment of hepatocellular carcinoma, gastric cancer and oral squamous cell carcinoma, the PIK3/AKT/mTOR pathway has an important role in the development of these cancers. By inhibiting this pathway, Salvianolic acid B can exert effective anti-tumour effects. In addition, in the treatment of non-small cell lung cancer and glioblastoma, salvianolic acid B inhibits the growth and differentiation of tumour cells by modulating the MAPK pathway to cut off intracellular signaling. The similarity of the treatment mechanisms of different cancers has implications for the treatment of cancer.

## Conclusion

Cancer is a malignant disease that affects human health and currently, various methods are available to slow down or stop cancer progression, such as surgery, chemotherapy, radiotherapy, and immunotherapy. However, these methods are associated with limitations such as tumor drug resistance and the specificity of the tumor location. In recent years, increasing attention has been paid to Chinese medicine in health protection, prevention, and treatment of diseases, as Chinese medicines can prevent the occurrence and development of various malignant diseases. *Salvia miltiorrhiza* Bunge (Lamiaceae) has been widely used in Chinese medicine for over 2000 years and contains several chemical constituents with unique biological effects. In particular, Sal-B induces apoptosis in cancer cells, such as lung, liver, stomach, glioma, and breast cancers by promoting ROS production and regulating energy metabolism but its exact effects on cancer need to be further investigated *in vivo*. Elucidating the correlation between Sal-B targets and its role in regulating energy metabolism homeostasis will facilitate further research on its antitumor mechanism, thus providing a scientific basis for further clinical research and application.

## Abbreviations

Sal-B: salvianolic acid B; Sal-A: salvianolic acid A; TCM: Traditional Chinese medicine; HCC: Hepatocellular carcinoma; HNSCC: Head and neck squamous cell carcinoma; OSCC: Oral squamous cell carcinoma; ROS: Reactive oxygen species; PKM2: Pyruvate kinase isozyme typeM2; EMT: Epithelial-mesenchymal transition; RECK: Reversion-inducing cysteine-rich protein with Kazal motifs; STAT3: Signal transducer and activator of transcription 3; AKT: protein kinase B; mTOR: mammalian target of rapamycin; MDM2: murine double minute2; ERK: Extracellular regulated protein kinases; Bcl-2: B-cell lymphoma-2; Bcl-xl: B-cell lymphoma–extra-large; GM3: ganglioside M3; GCS: glucosylceramide; COX-2: Cyclooxygenase-2; EGFR: epidermal growth factor receptor; PGE2: prostaglandin (PG) E2; PARP: poly ADP-ribose polymerase; HIF-1α: Hypoxia-Inducible Factor 1-Alpha; VEGF: vascular endothelial growth factor; NETs: neutrophil extracellular traps.
